# The factor structure of the general health questionnaire (GHQ12) in Saudi Arabia

**DOI:** 10.1186/s12913-018-3381-6

**Published:** 2018-08-02

**Authors:** Ashraf El-Metwally, Sundas Javed, Hira Abdul Razzak, Khaled K. Aldossari, Abdurrahman Aldiab, Sameer H. Al-Ghamdi, Mowafa Househ, Mamdouh M. Shubair, Jamaan M. Al-Zahrani

**Affiliations:** 1 King Abdullah International Medical Research Center (KAIMRC)/College of Public Health and Health Informatics, King Saud Bin AbdulAziz University for Health Sciences, Mail Code 2350; P.O. Box 3660, Riyadh, 11481 Kingdom of Saudi Arabia; 2 Docent of Epidemiology, School of Health Sciences, University of Tampere, Tampere, Finland; 3 College of Public Health and Health Informatics, King Saud Bin AbdulAziz University for Health Sciences, Riyadh, Saudi Arabia; 4 Ministry of Health and Prevention, Dubai, UAE; 5 Department of Family Medicine, College of Medicine, Prince Sattam Bin Abdulaziz University, Al-Kharj, Saudi Arabia; 6 Internal Medicine Department, College of Medicine, King Saud University, Riyadh, Saudi Arabia; 7 Department of Family Medicine, College of Medicine, Prince Sattam Bin Abdulaziz University, Al-Kharj, Saudi Arabia; 8 King Abdullah International Medical Research Center (KAIMRC)/College of Public Health and Health Informatics, King Saud Bin AbdulAziz University for Health Sciences, Riyadh, Saudi Arabia; 9 School of Health Sciences, University of Northern British Columbia (UNBC) 3333 University Way, Prince George, BC V2N 4Z9 Canada; 10 Department of Family Medicine, College of Medicine, Prince Sattam Bin Abdulaziz University, Al-Kharj, Saudi Arabia

**Keywords:** Psychological health, Saudi Arabia, General health, Exploratory (EFA) factor analysis, Al Kharj, General health questionnaire, GHQ-12

## Abstract

**Background:**

The General Health Questionnaire-12 (GHQ-12) is one of the most unique and extensively used self-report instruments for evaluating psychological disorders and strains. However, the factor structure of GHQ-12 has not been fully explored. The current study aims to assess the factorial structure of GHQ-12 in a large cross-sectional data-set extracted from Al Kharj central region of Saudi Arabia.

**Methods:**

Population based cross sectional data was extracted from January 2016 to June 2016 from Al Kharj population recruiting 1019 respondents aged 18 and above. Exploratory factor analysis (EFA) was applied together with multiple regression analysis to extract and retain factors. Mean GHQ-12 score for demographic and health-related traits were used for assessing this association. Statistical analysis was carried out using STATA version 12.1.

**Results:**

Three factors, including social dysfunction, anxiety, and loss of confidence were extracted from the factor structure. 55% of the overall variance was obtained through these factors. Total score of GHQ-12 ranged from 0 to 32 with a mean score of 12.

**Conclusion:**

Investigation of the factor structure of GHQ-12 demonstrated that GHQ-12 is a good measure for evaluating the general health of Saudi population. Future studies based on a larger sample size of non-clinical respondents will be useful to evaluate the practical effectiveness of GHQ-12 factors.

## Background

Psychological health is a major component contributing to overall well-being. The World Health Organization (WHO) [1] defined mental health “as a state of well-being in which every person realizes his or her own potential, can cope with the normal stresses of life, can work productively and fruitfully, and is able to contribute to her or his community.” By tradition, healthcare clinicians can successfully assess the state of an individual’s well-being by gauging substance abuse, anxiety, distress, and depression [2]. Therefore, mental health is not merely an absence of declared negative issues, but it is defined as a state of complete physical, mental, and social well-being [3]. Several instruments are being applied to examine the general health of an individual, but one of the most extensively used self-reported questionnaire consists of the general health questionnaire (GHQ) formulated by Goldberg in the 1970s and noted for being a reliable measure of mental health. The GHQ helps to screen psychological disorders in primary healthcare and outpatient settings [4–6] and has been utilized in cross-cultural settings [7, 8]. The GHQ has been used for both population based studies and health assessment surveys [9]. It is straightforward to administer, and can be completed in less than 10 min by a single participant [10].

The original GHQ is comprised of 60 items and is composed of a number of different versions such as GHQ-1, GHQ-12, GHQ-20, GHQ-28 and GHQ-30. From these listed versions, GHQ-12 is one of the most commonly used because of its ease of use [11, 12]. The GHQ-12 self-reported questionnaire includes 12 items, each of which is assessed through 4 indexes. Two of the most common types of scoring includes Likert scoring technique (0–1–2-3) and the bi-modal (0–0–1-1) [10]. Banks [13] suggested its usage for the GHQ-12 to compare levels of psychiatric impairment within and between samples. In different segments of the populations across countries, psychometric properties of this questionnaire in a variety of studies has also been appraised [14–18]. The GHQ-12 tends to have good specificity, reliability, and reasonably high sensitivity [19, 20]. Since its development by Goldberg [9], the GHQ has now being used in many different countries/cultures and has been translated into 38 languages; a testament to the validity and reliability of the questionnaire [21–25]. Other features that have not been fully explored on the GHQ-12 is the factor structure that underlies the instrument responses. Even though, this questionnaire was initially developed as a unidimensional scale, only limited studies were conducted using the GHQ-12 which provide empirical support to its one-factor latent structure [13, 26]. Alternatively, other multidimensional models such as 2 or 3 factors, are projected to be more suitable. As a result, a three-factor model, projected by Graetz in 1991, has much empirical support behind it [27–31]. These three factors include GPH-1 (social dysfunction & anhedonia; 6 items), GPH-2 (depression & anxiety; 4 items), and GPH-3 (loss of confidence; 2 items). A typical argument in favor and against the use of these models relate to the unidimensional solution that has repeatedly demonstrated a higher correlation between these factors. For instance, the correlation between the 3 factors varied from 0.72–0.84 in Padrón et al. [29], from 0.76–0.89 in Campbell and Knowles [28], and from 0.83–0.90 in Gao et al. [31]. Conversely, another argument applied against the model proposed by Graetz’s demonstrated a low discriminant validity concerning the factor scores i.e. a derivative of this model.

Considering the fact that factor analysis of the GHQ-12 has yielded two or three factor solutions. Two factor structures in an Iranian study [10] were similar to findings reported in the WHO study on psychological disorders in general healthcare [32]. Another study in New Zealand [33] supported the two-factor structure property of the 12 items GHQ, whereas, in a Spanish population [34] a three-factor structure was shown demonstrating successful stress, self-esteem, and coping. Rajabi et al. [35] indicated that three and two factor models of GHQ, fitted the data better than the one-dimensional model. Similarly, Gao [36] found that Graetz 3- factor model including Anxiety and Depression, Social Dysfunction, and Loss of Confidence fit the data better than other models. The aforementioned studies show the sample was demographically diverse including people from different education levels, general populations, industrial workers, and 18-year-old youth.

Despite being common, mental illness is underdiagnosed by health professionals. The reported prevalence of psychiatric or mental health disorders in Saudi Arabia vary from study to study [37]. Several studies have used GHQ-12 in a community setting in Saudi Arabia [38] however, there appears to be no evidence on the usefulness of GHQ-12 factor or if they exist in revealing between-patient difference in health-related quality of life and clinical states. Even though there are 3 identified distinct factors, it is usually difficult to differentiate them in clinical practice because all these factors are somehow correlated, which has been addressed by Gao et al. [36]. The study indicated that three factor domains of GHQ-12 fails to provide any supplementary information on psychological functioning of people in accordance to the health-related quality of life and clinical variables as compared with one dimensional measure. Whereas, homogeneity of the study population and small sample size, primarily coming from the clinical cases, may limit the generalizability of the study results. An in-depth study of factor structures would be valuable [39] because certain psychological domains can be a focus for targeting interventions to prevent further psychological deterioration within the population. Furthermore, in Saudi Arabia, to date, only one study successfully assessed the psychometric properties of the Arabic version GHQ-12 in university students [20]. However, no exploratory factor analysis (EFA) has been performed to assess the GHQ-12 factor structure. Therefore, verification and assessment of GHQ-12 factor structure is essential. Considering the different outcomes of the GHQ-12 factor structures in previous studies, this study was designed with the main objective to assess the factorial structure of GHQ-12 in a large sample recruited from Al Kharj central region in Saudi Arabia.

## Methods

### Study design and sample selection

Population based cross sectional survey was used in this study. Al Kharj, is locally renowned as “Al Saih”. It is one of the modern, stable and sophisticated city, and a Governorate in central Saudi Arabia, situated 77 km south of Riyadh with a population density of 376,000 where both urban and rural characteristics can be found. GHQ-12 responses were collected as a part of a larger study that investigated the main diabetic prevalence of 1019 participants in Al Kharj population [40]. Around 1003 (628 (62.61%) female and 375 (37.39%) male) out of 1019 had completed GHQ-12 responses, providing a high response rate of 98.4%.

### Inclusion and exclusion criteria

The study design is a secondary analysis of a population-based database of 1019 Al Kharj adult population (18 years of age and older). The proportion of individuals with diabetes was 4.3% (*n* = 43). The study included Saudi citizens, age18 years or above. Marital status, education, gender, smoking status, age, body mass index (BMI), and number of existing physical health issues were included as “individual-level characteristics”. The study excluded non-Saudi residents, Saudi citizens being younger than 18 years, or individuals not willing to take part in the study.

### Data collection

Psychological well-being of the participants was measured using the “12-item version of the General Health Questionnaire” (GHQ-12). Data were drawn from January 2016 to June 2016 with an overall sample size of 1019 participants in Al Kharj population. Respondents completed the questionnaires under the guidance of field workers. Surveyors did not influence the responses. Participants were free to respond openly to questions without fear of reprisal. After excluding incomplete questionnaires, which were defined as any questionnaire with missing responses to more than 5 questions, a total of 1003 questionnaires were returned, creating a response rate of 98.4%.

### Sampling technique

A multi-stage sampling technique was used. Samples from different governmental as well as private institutes were selected by means of a cluster sampling method. The total population of these institutions were divided into groups called clusters after obtaining a list of participants from each selected institute. Then samples of participants were selected using simple random sampling from each of the clustered group.

### Materials/instruments

Validated GHQ-12 for Arab population [20, 41] contains an equal number of positive and negative items and Likert method of scoring was used for rating scale in this study. Likert method of scoring ranges from 0 to 3: where zero represents the healthiest and 3 representing poor healthy/ illness and total score can range from 0 to 36. GHQ-12 questionnaire had four options for positive and negative items. For positive items, four options consisted of “Better than usual”, “Same as usual”, “Worse than usual” and “Much worse than usual” and were scored as 0, 1, 2 and 3 respectively. Whereas for negative items, four options consisted of “Not at all”, “Less than usual”, “Same as usual” and “More than usual” and were scored as 0, 1, 2 and 3 respectively.

### Data analysis

Descriptive statistics for demographic and health related characteristics were summarized using mean ± standard deviation and range for continuous data whereas, for categorical data using frequencies and percentage. All data were normally distributed before doing any statistical tests. Mean GHQ-12 score was computed and significance of differences was evaluated using one-way ANOVA with Tukey-karmer method or t-test for demographic and health related traits. To explore the factorial structure of Al Kharj population of GHQ-12, exploratory factor analysis (EFA) was achieved by means of principal factor analysis (PCA) to extract factors. The number of factors to be retained for PCA was assessed using scree plot and eigenvalue of more than 1.

Kaiser Normalization technique and varimax rotation (orthogonal rotated factor loadings) was used to interpret factor structure. Cut-off of absolute value 0.5 was used for factor loadings to be labeled for meaningful retained factors. “Bartlett test of sphericity” and “Kaiser-Meyer-Olkin (KMO)” measure of sampling adequacy was assessed prior to carrying out the PCA for suitability purposes. Additionally, multiple regression analysis was carried out for retained factor and mean GHQ-12 score to assess the relationship between demographic and health-related traits (social dysfunction, anxiety and loss of confidence). *P*-value of less than 0·05 indicated statistical significance and statistical analysis was carried out using STATA version 12.1.

### Ethics approval

Ethical approval was acquired from the local Institutional Review Board namely “Committee of Scientific Research and Publication.” Written informed consent was attained from all members aged ≥18 years. All data received from different participants were kept confidential.

## Results

### Demographic and health related traits

Demographic and health related traits for Al Kharj study sample are shown in Table 1. The study sample age ranged from 18 to 67 with a mean age of 26.36 and standard deviation of 8.63: where age structure consisted of 59.6% of 18–24 age group; 34.8% of 25–44 age group and 5.6% of 45–67 age group. There were 628 (62.61%) female and 375 (37.39%) male; 34.8% married and 65.20% single; and 82.15% university or highly educated respondents. Most participants were non-smokers (*n* = 873; 87.0%). There were 101 participants who were current smokers (10.1%) and only 29 (2.89%) participants considered ex-smokers. In this study sample of 130 ex- and current smokers, average smoking years were 8.28 with standard deviation 6.53 years. The average smoking frequency was 3 packs per day (SD = 1.89) for ex- and current smokers. Study sample comprised mostly of 18–44 years, single, highly educated, female and non-smokers.

**Table 1:** Demographic and health related characteristics of Al Kharj population

	Mean (SD) Or Frequency (Percentage)^a^	Range
Age (years)	**26.36 (8.62)**	(18–67)
18–24	598 (59.6)^a^	
25–44	349 (34.8)	
45–67	56 (5.6)	
Gender		
Female	628 (62.61)	
Male	375 (37.39)	
Marital Status		
Married	349 (34.8)	
Single	654 (65.20)	
Education Level		
Primary	22 (2.19)	
Secondary	128 (12.76)	
Intermediate	29 (2.89)	
University Level	788 (78.56)	
Postgraduate	36 (3.59)	
Smoking Status		
Current	101 (10.07)	
Ex-smoker	29 (2.89)	
Non-Smoker	873 (87.04)	
Smoking years- (N = 130)	**8.28 (6.53)**	(0–30)
Smoking Frequency (Packs per day) - (*N* = 130)	**3.19 (1.89)**	(1–11)
Diabetes		
Yes	43 (4.29)	
No	960 (95.71)	
Body Mass Index (BMI)	**26.81 (6.66)**	(15.02–57.22)
Normal (18.5–24.9 kg/m^2^)	459 (45.76)	
Overweight (25.0–29.9 kg/m^2^)	267 (26.62)	
Obese (≥ 30 kg/m^2^)	277 (27.62)	
Waist Circumference (WC)	**84.39 (18.60)**	(48.5–180)
Chronic diseases		
Yes	82 (8.18)	
No	921 (91.82)	
Chronic disease Type- (N = 82)		
Diabetes	43 (52.4)	
Hypertension	32 (39.02)	
Both	7 (8.54)	
Psychological Disease		
Yes	53 (5.28)	
No	950 (94.72)	
Psychological Disease Type- (N = 53)		
Anxiety	21 (39.6)	
Depression	9 (17.0)	
Unknown	23 (43.4)	
Chronic pain		
Yes	191 (19.04)	
No	812 (80.96)	
Total GHQ score	**12 (5.23)**	(0–32)
−25% percentile	8	
−50% percentile (Median)	11	
−75% percentile	16	

^a^Highlighted numbers in this column are mean (SD) and highlighted items are frequencies and percentages

Body mass index (BMI) ranged from 15 to 57 kg/m^2^ with an average BMI found to be 26.81 (SD = 6.66). Overweight status was defined by a BMI of 25.0–29.9 kg/m^2^, and obesity was defined has having a BMI of ≥30 kg/m^2^. Chronic pain was a dichotomous question simply asking respondents whether they have chronic pain (yes/no). Likewise, the presence of psychological disease was based on self-reported dichotomous responses (yes/no).

Waist circumferences ranged from 48.5–180 cm in the study population. Participants with normal body mass index were 45.8% (*n* = 459), 26.6% overweight (*n* = 267) and 27.6% obese (*n* = 277). Participants with different health related traits and outcomes were; 4.3% (*n* = 43) clinically confirmed diabetic participants, 8.2% (*n* = 82) had chronic disease of which 43 were diabetic, 32 hypertension and 7 had both. Also 5.3% (*n* = 53) had psychological disease; 21 of these had anxiety, 9 had depression and 23 reported unknown. 81% (*n* = 812) of the participants reported having chronic pain which consisted from one pain site to multiple pain sites such as the arm, leg, hand, wrist, back and chest pain.

### GHQ-12 and mean GHQ-12 score

**Fig. 1:**
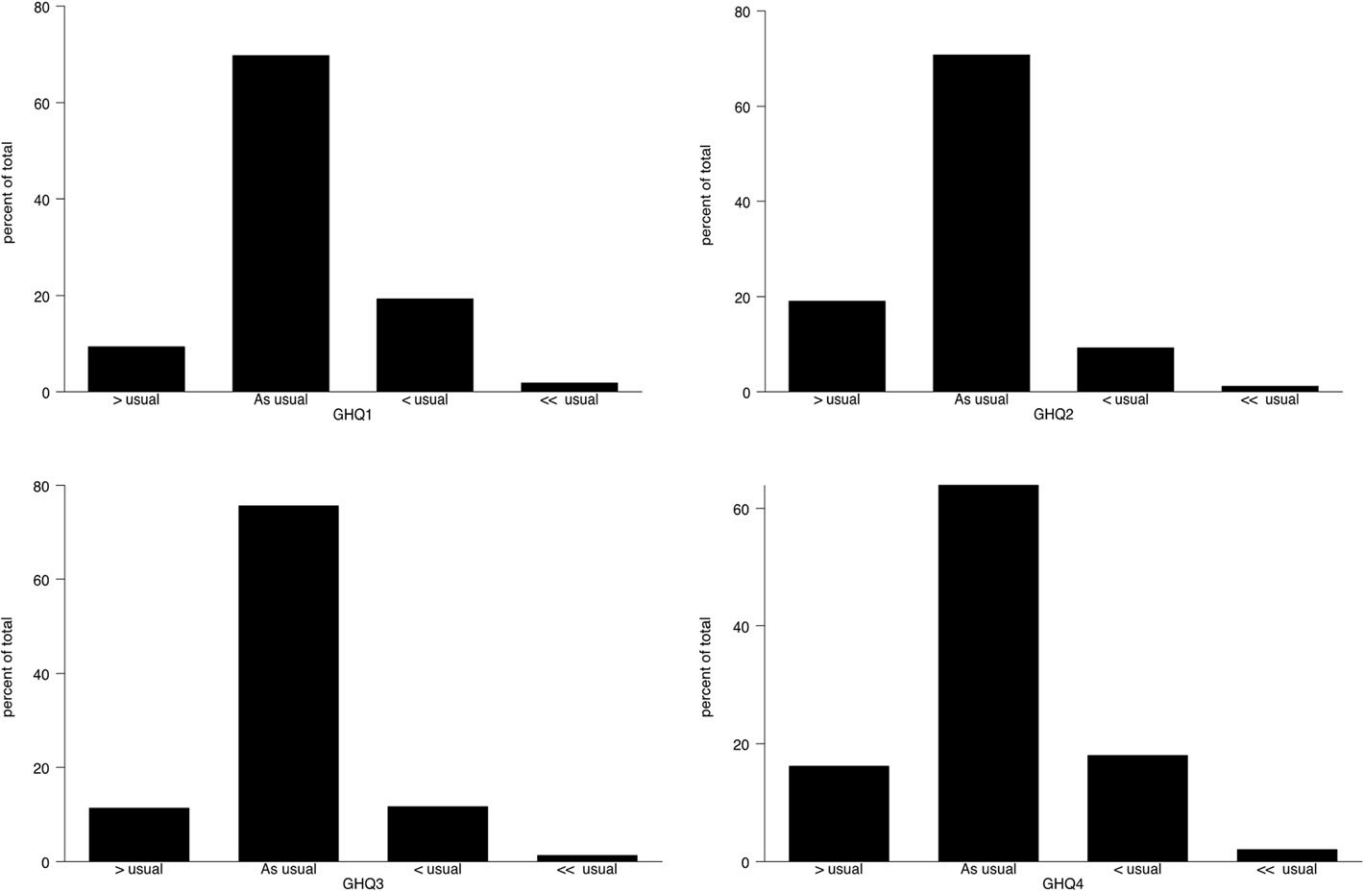
Distribution for 1–4 items of the General health questionnaire

**Fig. 2:**
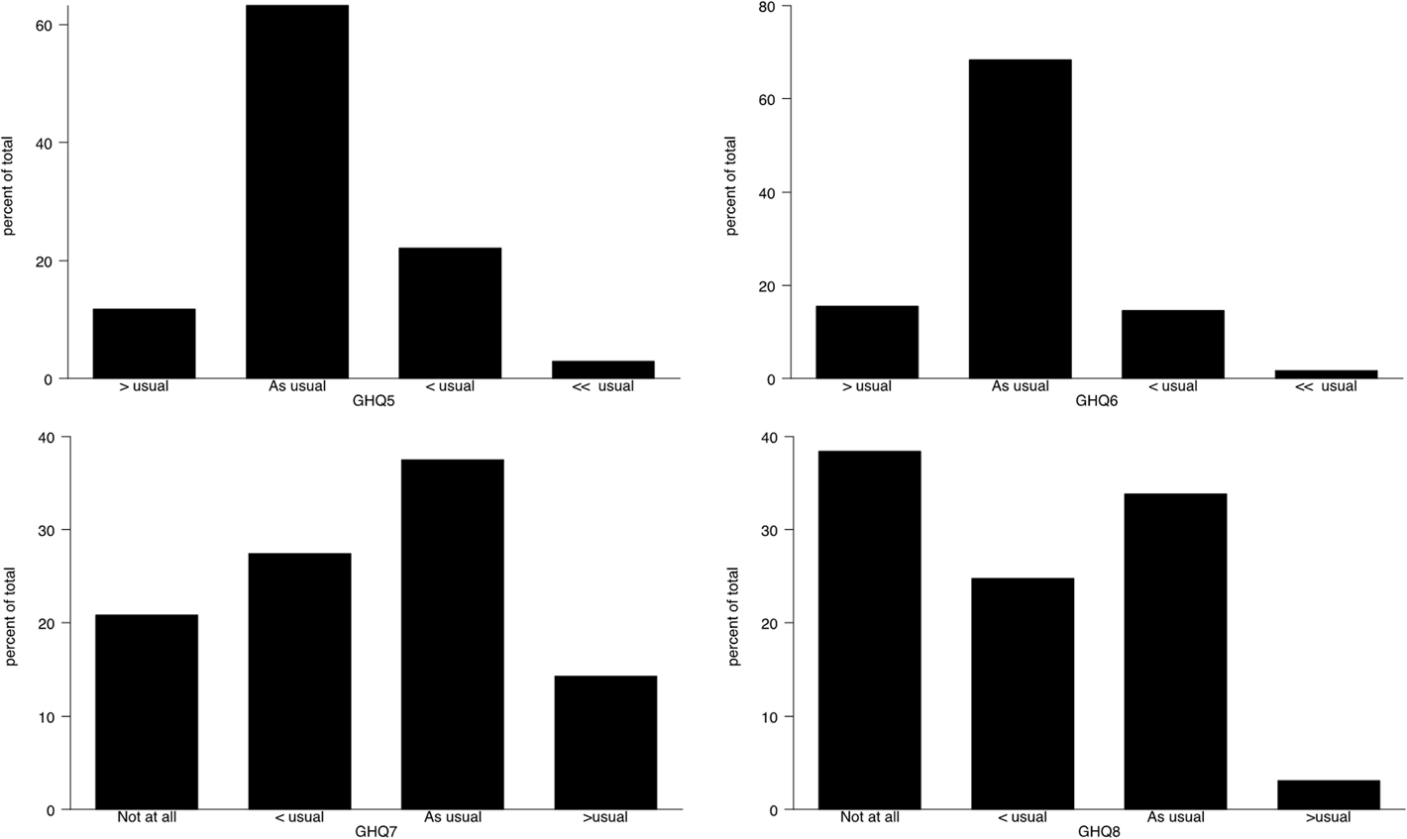
Distribution for 5–8 items of the General health questionnaire

**Fig. 3:**
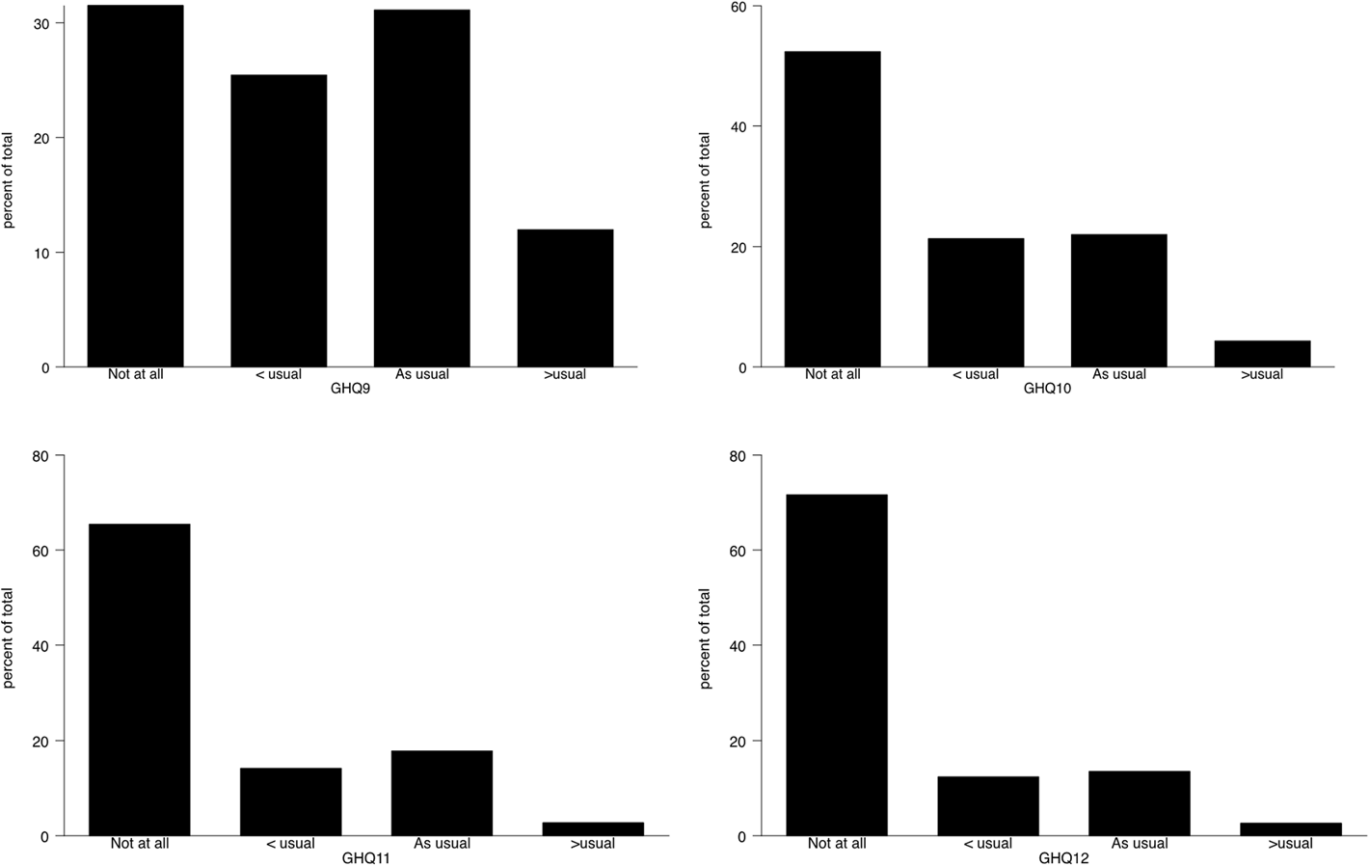
Distribution for 9–12 items of the General health questionnaire

GHQ-12 is comprised of 6 positive and 6 negative items to assess positive and negative mental health. For our study population, wording of GHQ-12 questionnaires with their rating scale coding is shown in Table 2. The median was used to assess central tendency for GHQ-12 questionnaires, where all the positive items had a median score of 1 which represents the “same as usual” response. But negative items had a different median score for each questionnaire; GHQ-7 had median score of 2 represents “same as usual” response, GHQ (8, 9) had median score of 1 represents “Less than usual” response and GHQ (10, 11 and 12) had median score of zero represents “Not at all”. This can be visualized clearly through Figs. 1, 2 and 3 which indicates the most frequent and location of the response for each questionnaire item. Higher Score on the rating scale indicates poor mental health and wellbeing.

**Table 2:** Description of GHQ coding and GHQ-12 descriptive statistics for Al Kharj Population

	Questionnaire Item	Item coding and Type	Median
GHQ-1	Have you recently been able to concentrate on whatever you’re doing?	Positive [0,1,2,3]*	1
GHQ-2	Have you recently felt that you were playing a useful part in things?	“	1
GHQ-3	Have you recently been feeling reasonably happy, all things considered?	“	1
GHQ-4	Have you recently felt capable of making decisions about things?	“	1
GHQ-5	Have you recently been able to enjoy your normal day-to-day activities?	“	1
GHQ-6	Have you recently been able to face up to problems?	“	1
GHQ-7	Have you recently felt constantly under strain?	Negative [0,1,2,3]*	2
GHQ-8	Have you recently felt you couldn’t overcome your difficulties?	“	1
GHQ-9	Have you recently lost much sleep over worry?	“	1
GHQ-10	Have you recently been feeling unhappy or depressed?	“	0
GHQ-11	Have you recently been losing confidence in yourself?	“	0
GHQ-12	Have you recently been thinking of yourself as a worthless person?	“	0

*Likert method coding as zero representing most healthy and 3 representing poor healthy/ illness

For the study population, “total GHQ-12 score” ranged from 0 to 32 with mean score of 12 and standard deviation 5.23; 25% of participants had total GHQ score 8 or less, 50% of participants had total GHQ score of 11 or less whereas 75% of participants had total GHQ score of 16 or less. Significant differences between different group levels for total GHQ-12 score was evaluated using Anova with Tukey-karmer or t-test for different traits. Females had a mean total GHQ-12 score of 12.22 (±5.30) whereas males had 11.26 (±5.04). Females and males had significant differences in total GHQ-12 score, as indicated by t-test statistics of 2.85 (DF = 1001) and *p*-value of 0.0045 at 5% significance level. Mean of total GHQ-12 score was 11.99 (±5.13) for 18–24 years, 11.73 (±5.43) for 25–44 years and 11.43(±4.92) for 45–67 years; indicating decrease in mean total GHQ score.

Mean of total GHQ-12 score for chronic disease absence was 11.77 (SD = 5.22) vs presence of chronic disease 12.98 (SD = 5.19). This difference of − 1.21 for chronic pain in mean total GHQ-12 score (t-test − 2.01, *p*-value 0.045) was found to be statistically significant at a 5% significance level. Similarly, mean of total GHQ-12 score for psychology diseases (such as anxiety, and depression) absence was reported to be 11.64 (SD = 5.13) vs presence mainly reported to be 15.96 (SD = 5.24). This difference of − 4.32 for psychology disease in mean total GHQ-12 score (t-test − 5.96, *p*-value < 0.001) was found to be statistically significant at 5% significance level. We also found significant difference in mean total GHQ-12 score of − 1.84 for chronic pain (t-test − 4.42, p-value < 0.001) at 5% significance level; where total GHQ-12 score mean for chronic pain presence was 13.36 (SD = 5.01) and for absence of chronic pain was 11.52 (SD = 5.22).

### GHQ-12 factor analysis

Factor analysis suitability was tested using KMO value of 0.84 which surpassed the recommended value of 0.6 and Bartlett Test of sphericity (chi2 = 2926.85, DF = 66, p-value< 0.001) was found to be statistically significant at 0.05% significance level. To scrutinize factorial structure of Al Kharj population of GHQ-12, exploratory factor analysis (EFA) was achieved by means of the principal factor analysis, Kaiser Normalization and varimax rotation for factor extraction. Three factors were extracted based on eigenvalue of more than 1 criterion and inspection of scree plot and all 3 factors account for 55% variance of the GHQ-12 total variance. GHQ-12 items 1 to 6 had factor loading of more than 0.5 for factor 1, GHQ-12 items 7 to 10 had factor loadings more than 0.5 for factor 2 whereas GHQ-12 items 11 and 12 had factor loadings more than 0.5 for factor 3 as shown in Table 3. Factor one was labeled social dysfunction, factor two labeled as anxiety and factor 3 as loss of confidence.

**Table 3:** Principal component Factor analysis (Kaiser Normalized and rotated factor loadings) and its variance structure of 12 item General Health Questionnaire for Al Kharj Population

	Factor 1 (Social Dysfunction)	Factor 2 (Anxiety)	Factor 3 (Loss of confidence)
Eigenvalue	2.57	2.09	1.94
Variance Explained (%)	21	18	16
GHQ-1	0.66		
GHQ-2	0.63		
GHQ-3	0.57		
GHQ-4	0.73		
GHQ-5	0.64		
GHQ-6	0.63		
GHQ-7		0.78	
GHQ-8		0.61	
GHQ-9		0.68	
GHQ-10		0.57	
GHQ-11			0.83
GHQ-12			0.83

A blank represents absolute loadings less than 0.5

### Association with GHQ-12 factors

After checking for all assumptions of multivariable (multiple) linear regression analysis, we carried out a multiple linear regression analysis for the retained (extracted) three factors (social dysfunction, anxiety and loss of confidence) for demographic and health-related traits to assess the relationship as shown in Table 4.

**Table 4:** Associations of demographic and health related characteristics with GHQ-12 factors

	Factor 1 (Social Dysfunction)		Factor 2 (Anxiety)		Factor 3 (Loss of confidence)	
	Beta (S.E)	*P*-value	Beta (S.E)	*P*-value	Beta (S.E)	*P*-value
Age (years) (v. 18–24 years)						
25–44	− 0.26 (0.70)	< 0.001	0.16 (0.07)	0.019	0.01 (0.06)	0.882
45–67	− 0.54 (0.14)	< 0.001	0.16 (0.14)	0.242	0.18 (0.14)	0.189
Gender (v. Male)						
Female	0.30 (0.6)	< 0.001	0.04 (0.06)	0.578	−0.03 (0.06)	0.666
Diabetes (v. No)						
Yes	−0.6 (0.16)	0.678	−0.02 (0.15)	0.891	0.03 (0.15)	0.867
Body Mass Index (BMI) (v. Normal)						
Over- weight	−0.23 (0.08)	0.003	0.02 (0.07)	0.758	0.04 (0.08)	0.604
Obese	−0.19 (0.08)	0.011	0.12 (0.08)	0.117	0.15 (0.08)	0.052
Waist Circumference (WC)	−0.01 (0.01)	< 0.001	0.003 (0.01)	0.126	0.002 (0.01)	0.295
Marital Status (v. Single)						
Married	−0.27 (0.7)	< 0.001	0.05 (0.07)	0.432	0.06 (0.07)	0.330
Education Level (v. Primary)						
Secondary	0.17 (0.22)	0.448	0.43 (0.23)	0.062	−0.57 (0.23)	0.013
Intermediate	0.06 (0.28)	0.824	0.32 (0.28)	0.250	−0.78 (0.28)	0.006
University Level	0.42 (0.21)	0.050	0.29 (0.21)	0.172	−0.88 (0.21)	< 0.001
Postgraduate	0.10 (0.27)	0.709	0.59 (0.27)	0.027	−0.77 (0.27)	0.004
Smoking Status (v. Non- Smoker)						
Ex-smoker	−0.05 (0.18)	0.794	0.03 (0.19)	0.891	−0.52 (0.19)	0.006
Current Smoker	−0.14 (0.11)	0.172	0.04 (0.11)	0.670	0.01 (0.10)	0.945
Smoking years- (N = 130)	−0.02 (0.01)	0.03	8 × 10^-4 (0.01)	0.921	−0.008 (0.01)	0.345
Smoking Frequency (Packs per day)	−0.02 (0.03)	0.449	5 × 10^-4 (0.03)	0.987	−0.03 (0.03)	0.405
Chronic diseases (v. No)						
Yes	−0.16 (0.11)	0.155	0.35 (0.11)	0.003	0.19 (0.12)	0.098
Chronic disease Type- (v. Diabetes)						
Hypertension	−0.08 (0.26)	0.773	0.54 (0.25)	0.039	0.12 (0.33)	0.724
Psychological Disease (v. No)						
Yes	0.58 (0.14)	< 0.001	0.45 (0.14)	0.001	0.44 (0.14)	0.002
Psychological Disease Type- (v. Anxiety)						
Depression	0.29 (0.53)	0.583	0.16 (0.42)	0.705	0.55 (0.55)	0.329
Chronic pain (v. No)						
Yes	0.20 (0.08)	0.011	0.33 (0.08)	< 0.001	0.05 (0.08)	0.533

Age groups were negatively associated with social dysfunction score for all age group levels; where 25–44 year group had − 0.26 (S.E = 0.70, *p*-value< 0.001) and 45–67 year group had − 0.54 (S.E = 0.14, p-value< 0.001) compared to the 18–24 year age group. Only age group 25–44 (Beta = 0.16, S.E = 0.07, p-value< 0.019) showed positively and statistically significantly associations with anxiety factor but not for the loss of confidence factor. Female gender was positively significantly associated with social dysfunction score (Beta = 0.3, S.E = 0.6, p-value< 0.001) compared to male but not for anxiety or loss of confidence factors. Similarly, BMI, waist circumference, marital status and smoking years were significantly and negatively associated with social dysfunction. But, psychological disease and chronic pain was positively and significantly associated with social dysfunction. Chronic disease, chronic disease type, psychological disease and chronic pain were positively and statistically significantly associated with anxiety factor. Additionally, postgraduate level education (vs primary education level) was significantly and positively associated with anxiety factor. We found that education level and current smoker (vs non-smoker) were negatively and statistically significantly associated with loss of confidence factor. However, chronic disease type and psychological disease were positively and statistically significantly associated with loss of confidence factor.

## Discussion

To our best knowledge, this is the first study to assess the factorial structure and psychometric properties of the GHQ-12 in males and females using a study sample from Al Kharj central region. Exploratory Factor Analysis (EFA) was used to identify the potential factor structure of GHQ-12. However, EFA did not allow model assessment as identifications of factors are based on the arbitrary cut-points, for which the criteria for goodness-of-fit cannot be used. Li et al. [42] found similar findings in the context of the GHQ-12.

In our study, the PCA extracted 3 factors (Factor 1, Factor 2, and Factor 3) from the GHQ-12. Factor one was labeled as social dysfunction (items 1–6), factor two was labeled as anxiety (items 7–10) and factor 3 as loss of confidence (items 11–12); based on previous literature review and absolute value of 0.5 factor loading for each GHQ item represents the factor labeling. The findings from exploring the factor structure of GHQ-12 were similar to those of several other international versions [29, 43]. These 3 factors explained 55% of the overall variance. Our findings were similar to that of previous factor analytic studies of GHQ-12 that have found three-factor solution [20, 35, 44]. In conformity, Martin and Newell [45] in their factor analysis of Graetz three-factor solution utilizing likert scoring revealed three factors: Anxiety, social dysfunction, and loss of confidence. As shown in table three, the factor loadings were represented to be high in our study.

By studying a large sample of 6151 young Australians, aged 16–25 years in a longitudinal and cross-sectional analysis, Graetz [27] acknowledged that GHQ-12 structure included 3 distinct dimensions, anxiety/depression, low social function and loss of confidence. The corresponding elucidated variances were roughly about 37.0, 12.0 and 8.0%. The findings were supported and verified by several other studies. Some of the research studies for example, Martin [44], and Daradkeh [20] revealed that the GHQ-12 was 3-dimensional, nonetheless, it utilized wide-ranging names from those proposed and used by Graetz [27]. In our study, factors designated as anxiety, social dysfunction, and loss of confidence, were subject to variation possibly due to being more under the influence of the socio-cultural norms and values that govern the population under study. Our study was conducted in Al Kharj and not in other regions, generalizing our results to Saudi Arabia might be of question, however, the characteristics of the population of Al Kharj is comparable to that of all Saudi Arabia, which is mixed between urban and rural population. The association between GHQ scores and education was reported to be non-significant in our study which is consistent with the study conducted by Banks, 1982 [46] - except for intermediate class and loss of confidence. This was because of the opposite trends as people having higher level of education were more prone towards social dysfunction and anxiety, while being less prone to lack of confidence.

This study included respondents mostly within an age range of 18–44 age group, single, females, highly educated, and non-smokers. Our results showed that married participants had better mental health than individuals who were single. This finding corresponds with the findings of the Saki and Kaikhavani [47] study. On the other hand, Maleki et al. [48] demonstrated that mental disorders among women increase with age. Therefore, the variation between the mean total GHQ-12 score was considerably non-significant between age groups and other two factors, while it was statistically significant for social dysfunction. In our study, females were significantly associated with scores obtained for social dysfunction, but not for anxiety or loss of confidence factors as compared to males. In our study, females were comparably related to the presence of higher rates of negative mental health items.

### Strengths and limitations

The major strength of the study was its large sample size having a variety of characteristics. This permitted substantial variation in the score obtained from GHQ-12, to facilitate the outcomes which can be extrapolated to a similar population. Moreover, GHQ12 has evidenced itself to be a valued measure of psychiatric disorders. The results obtained in our study were in line with the studies conducted previously on adult and young population.

This study has some limitations which must be pointed out. Molina [49] revealed that “wording effects” of the items needs to be controlled while analyzing the “GHQ-12 factor structure”, but such effects had not been appropriately examined in our study. Although the response rates were high, however, there was a possibility that the absence of the participants during the conduction of survey might be due to some health care issue that may affect the GHQ-12. Since, only 1.6% of the respondents did not participate, it could have only left a minimal impact on the outcomes of the GHQ-12 psychometric characteristics. Moreover, a cross sectional study design used in the study limits causal inferences.

### Implications and recommendations

The availability and applicability of a reliable and valid instrument like the one used in our study (GHQ-12) is critical for health professionals to recognize those who are at a greater risk of suffering from mental health issues, with an aim of promoting mental health interventions that can be planned, implemented and evaluated properly. Future research studies with non-clinical population-based larger sample size will be useful to additionally evaluate the practical effectiveness of the GHQ-12 factors.

## Conclusion

Our results help us to confirm the factorial structure of GHQ-12 in a population based sample of Al Kharj, Saudi Arabia. This tool has been validated for several population thus, being translated into different languages. We conclude that in the Saudi citizens, (age range from 18 and above) from Al Kharj, Saudi Arabia, the GHQ-12 has shown very good psychometric characteristics and supports the scale applicability in this population. Furthermore, the study revealed that item factors were loaded into three main components using principal components analysis including social dysfunction, anxiety, and loss of confidence presenting an overall variance of 55%.

## Abbreviations

BMI: Body Mass Index
EFA: Exploratory factor analysis
GHQ-12: General Health Questionnaire-12
GPH: General Psychological Health
KMO: Kaiser-Meyer-Olkin
